# Microstructures, Mechanical Properties, and Corrosion Behavior of As-Cast Mg–2.0Zn–0.5Zr–xGd (wt %) Biodegradable Alloys

**DOI:** 10.3390/ma11091564

**Published:** 2018-08-30

**Authors:** Huai Yao, Jiuba Wen, Yi Xiong, Ya Liu, Yan Lu, Wei Cao

**Affiliations:** 1 School of Materials Science and Engineering, Henan University of Science and Technology, Luoyang 471023, China; xy_hbdy@163.com (Y.X.); liuya_021@163.com (Y.L.); luyan@haust.edu.cn (Y.L.); 2 Collaborative Innovation Center of Nonferrous Metals, Luoyang 471023, China; 3 Nano and Molecular Systems Research Unit, University of Oulu, Oulu FIN-90014, Finland; wei.cao@oulu.fi; 4 School of Mechanical and Automotive Engineering, Anhui Polytechnic University, Wuhu 241000, China

**Keywords:** magnesium alloy, microstructures, mechanical properties, corrosion, polarization

## Abstract

The Mg–Zn–Zr–Gd alloys belong to a group of biometallic alloys suitable for bone substitution. While biocompatibility arises from the harmlessness of the metals, the biocorrosion behavior and its origins remain elusive. Here, aiming for the tailored biodegradability, we prepared the Mg–2.0Zn–0.5Zr–xGd (wt %) alloys with different Gd percentages (x = 0, 1, 2, 3, 4, 5), and studied their microstructures and biocorrosion behavior. Results showed that adding a moderate amount of Gd into Mg–2.0Zn–0.5Zr alloys will refine and homogenize α-Mg grains, change the morphology and distribution of (Mg, Zn)_3_Gd, and lead to enhancement of mechanical properties and anticorrosive performance. At the optimized content of 3.0%, the fishbone-shaped network, ellipsoidal, and rod-like (Mg, Zn)_3_Gd phase turns up, along with the 14H-type long period stacking ordered (14H-LPSO) structures decorated with nanoscale rod-like (Mg, Zn)_3_Gd phases. The 14H-LPSO structure only exists when x ≥ 3.0, and its content increases with the Gd content. The Mg–2.0Zn–0.5Zr–3.0Gd alloy possesses a better ultimate tensile strength of 204 ± 3 MPa, yield strength of 155 ± 3 MPa, and elongation of 10.6 ± 0.6%. Corrosion tests verified that the Mg–2.0Zn–0.5Zr–3.0Gd alloy possesses the best corrosion resistance and uniform corrosion mode. The microstructure impacts on the corrosion resistance were also studied.

## 1. Introduction

The magnesium alloys have attracted considerable attentions in biomaterial applications, due to their outstanding mechanical properties and unique biodegradability in the physiological environments [1,2]. The Young’s modulus of magnesium alloys (41–45 GPa) is closer to that of human bone than these of Co–Cr alloys (230 GPa), stainless steels (189–205 GPa), Ti alloys (110–117 GPa) and synthetic hydroxyapatites (73–117 GPa). This can diminish stresses at implant/bone interface and stimulates new bone growth, resulting in better implant stability [3]. As an essential element in human body, the Mg element also benefits bone strength and growth [4]. Furthermore, it acts as a co-catalyst for enzymatic reactions, and synthesis and replication of RNA and DNA [5]. The Mg alloys are degradable in aqueous ambience, and the excessive Mg element is harmlessly excreted [6]. For the above reasons, they are ideal biological materials for temporary transplantation.

In spite of the aforementioned advantages, using Mg alloys, in vivo, suffer from fast degradation rates from the Mg matrices. Consequentially, mechanical integrity is deteriorated along with localized alkalization, and rapid release of corrosion products (e.g., hydrogen gas and large particles) [7]. To improve the corrosion resistance and mechanical properties, element alloying has been widely applied [8]. Typical alloying counterparts are selected among aluminum (Al), zinc (Zn), lithium (Li), yttrium (Y), zirconium (Zr), manganese (Mn), and rare earth elements (RE). However, only a few of them are functional in biomedical applications to satisfy the biocompatibility [9]. For instance, as an essential but harmless element in the human body, zinc provides strength for Mg alloy, due to solid solution strengthening [10]. Alloying the Zr with Mg will effectively refine the grain size and improve mechanical property and corrosion resistance [11]. Similar strengthening effects can also be achieved by using the Gd as an alloy element. Though with certain toxicity in living body, the Gd can be released below the harmful rates subjected to proper controls [12]. Following these progresses, several Mg–Zn–Zr–Gd alloys were developed [13]. In contrast to dedicated studies of mechanical properties after Gd addition [14], the corrosion behaviors are rather scarcely reported for the Mg–Zn–Zr–Gd alloys. 

In this work, we casted the Mg–2.0Zn–0.5Zr–xGd alloys with various Gd contents, and studied their microstructures, mechanical properties, and biocorrosion behaviors. Evolution of the microstructures and mechanical properties was revealed following the variation of the Gd content. The corrosion resistances were evaluated through the mass loss, hydrogen evolution, and potentiodynamic polarization under the immersion of the alloys in simulated body fluid (SBF). An optimized percentage was figured out, and impacts of microstructural evolution on mechanical properties and anticorrosion were studied.

## 2. Experimental Section

### 2.1. Sample Preparation

High purity Mg (purity ≥ 99.93%), high purity Zn (purity ≥ 99.93%), Mg–25%Zr, and Mg–20%Gd master alloys were employed to prepare the Mg–2.0Zn–0.5Zr–xGd (x = 0, 1, 2, 3, 4, 5) alloys. They were melted by electric induction furnace under the CO_2_ + 2%SF_6_ protection gas to prevent oxidizations of the Mg. The equivalent amount of master alloys were added when temperature reached approximately 710 °C. The melted mixture was further held for about 20 min at 730 °C, and then poured into a mild steel mold with size of 160 mm × 45 mm × 100 mm pre-heated to 300 °C.

### 2.2. Microstructure Determination and Mechanical Properties Test

The ingots were cut into small pieces for microstructure analysis. The microstructures were characterized using optical microscopy (OM, OLYMPUS PMG3, JAPAN) and scanning electronic microscopy (SEM, JSM-5610LV, JEOL, JAPAN) equipped with energy dispersive spectroscope (EDS, Phoenix, EDAX, USA) to conduct element analysis. Transmission electron microscopy (TEM) was carried out using a JEM-2100 (JEOL, JAPAN) at 200 kV. Thin foils for TEM observation were punched into discs of 3 mm in diameter, mechanically polished to approximately 70 μm, and then twin-jet electropolished in a solution of 97% ethyl alcohol and 3% perchloric acid at −45 °C and 0.1 A. Finally, the twin gun precision ion polishing system (model 691, Gatan, USA) was used for ion milling. The alloy ingots were machined into tensile specimens of 5 mm gauge diameter and 25 mm length. Tensile tests were performed at room temperature by using an AG-1250 KN (SHIMADZU, JAPAN) machine at a tensile speed of 1 mm/min.

### 2.3. Electrochemical Corrosion Test

The electrochemical experiments were carried out with an electrochemical workstation (Autolab PGSTAT128N, Metrohm, Switzerland) in SBF. The SBF consists of 8.0 g/L NaCl, 1.0 g/L glucose, 0.4 g/L KCl, 0.35 g/L NaHCO_3_, 0.14 g/L CaCl_2_, 0.1 g/L MgCl_2_·6H_2_O, 0.06 g/L Na_2_HPO_4_·12H_2_O, 0.06 g/L KH_2_PO_4_, and 0.06 g/L MgSO_4_·7H_2_O [15,16]. The three-electrode configuration was adopted in the electrochemical test. The alloy sample (Φ11.3 × 10 mm) was used as the working electrode, a saturated calomel electrode as the reference electrode, and a graphite sheet as the counter electrode. Potentiodynamic polarization curve test was performed at a scanning rate of 1 mV s^−1^ from −0.25 V in the cathodic direction to +0.4 V in the anodic direction based on the open circuit potential (OCP).

### 2.4. Immersion Corrosion Test

In the weight loss experiment, the loss Δ*W* (mg) can be translated to an average corrosion rate P_w_(mm/y) using

(1) $$P_{w}=87.6\Delta W/\rho At$$

Here, ρ is the alloys density (g/cm^3^), A the specimen surface area (cm^2^), and *t* the immersion time (h) [17]. A typical sample dimension is 18 mm diameter and 5 mm thickness. It was immersed in 180 mL SBF. The temperature was maintained at 37 °C. The immersion test lasted for 120 h, and the SBF was renewed every 8 h in order to keep a stable pH value.

The corrosion rate was also evaluated in SBF by a hydrogen evolution experiment. The sample with the typical dimension was horizontally immersed in 180 mL SBF, which was kept in a beaker open to air. The hydrogen evolved during the corrosion experiment was collected in a burette above the corroding sample. The amount of hydrogen can be measured from the height difference of the SBF in the measuring burette [18]. Samples were immersed in SBF for up to 120 h at 37 °C, three specimens for average. The hydrogen bubbles generated from the specimen were collected by funnel and burette, and the total volume of evolved hydrogen gas ($V_{H}$) was recorded during the time interval of the SBF renewal (every 8 h) in order to achieve precise results. The hydrogen evolution rate, $V_{H}$ (mL), was converted to corrosion rate [19], $P_{H}$ (mm/y) using

(2) $$P_{H}=95.36V_{H}/\rho At$$

## 3. Results and Discussion

### 3.1. Microstructures

**Figure 1:**
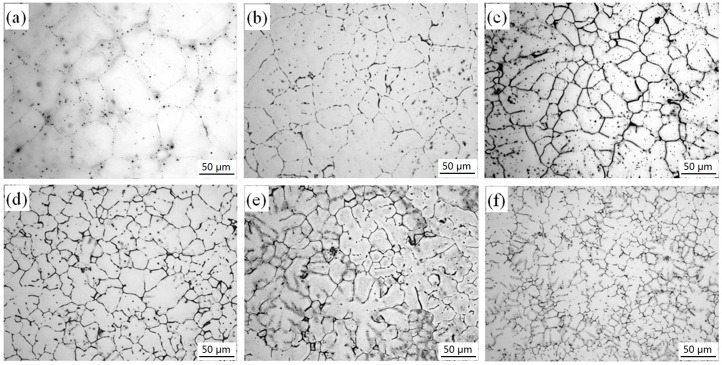
Optical micrographs of Mg–2.0Zn–0.5Zr–xGd alloys: (**a**) x = 0; (**b**) x = 1; (**c**) x = 2; (**d**) x = 3; (**e**) x = 4; (**f**) x = 5.

Figure 1 shows optical micrographs of as-cast Mg–2.0Zn–0.5Zr–xGd (hereafter xGd, with x referring to the percentage) alloys with varied Gd contents. For the one without Gd, the secondary phase is shown as a point distribution in α-Mg matrix. After adding Gd, the samples turn to white α-Mg matrix, and black secondary phases distributed along and/or adjacent to the grain boundaries as shown in Figure 1b–f. When the content of Gd was less than 3.0 wt %, the distribution of secondary phases was discontinuous, and part of the secondary phase particles stayed separately as grains. The amount of secondary phase particles increases with the Gd content, but the grain size decreases. When the content increased from 3.0 to 5.0 wt %, the secondary phase interconnects to form networks, and gradually grows into coarse and compact lines. The grain size of alloys continue decreasing with the Gd content. When Gd content was 1.0, 2.0, 3.0, 4.0, and 5.0 wt %, the average grain size of the alloy was about 42.4, 27.7, 25.1, 22.4, and 16.3 μm, respectively. During solidification, Gd element tends to accumulate at the front of the solid–liquid interface, which increases the supercooling phenomenon in the solid–liquid interface area, leading to the increase of nucleation rate. At the same time, Gd can also reduce the critical nucleation radius of magnesium alloy, making the alloy easy to nucleate, and promoting the grain refinement [20].

**Figure 2:**
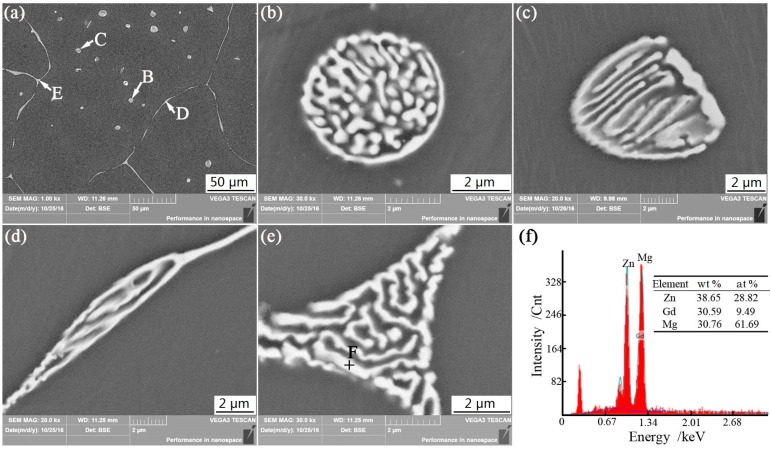
Microstructure of 3Gd alloy: (**a**) lower magnification; (**b**–**e**) higher magnifications of secondary phases; (**f**) results of EDS.

The general SEM micrograph of 3Gd alloy is presented in Figure 2a, with magnified SEMs at points B, C, D, and E, depicted in Figure 2b–e. The secondary phases are different in size and shape, as shown in the figures. While the small network ellipsoidal-shaped secondary phase mainly locates in the interior of the grains (Figure 2b,c), the network fishbone-shaped secondary phase tends to have a continuous distribution at grain boundary (Figure 2d,e). In Figure 2f, the EDS analysis of network fish-shaped secondary phase indicates the main compositions are the Mg, Zn, and Gd.

**Figure 3:**
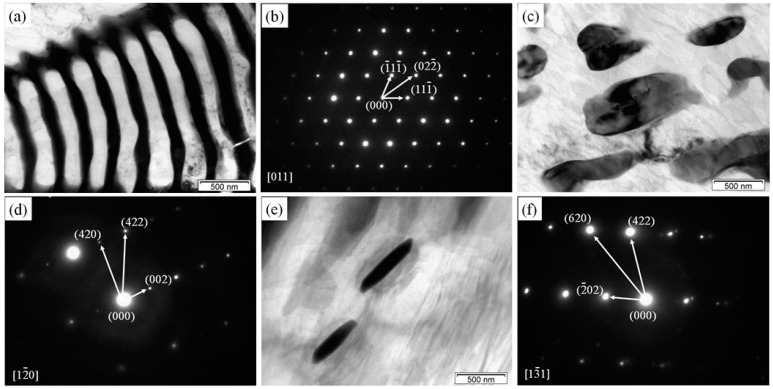
Microstructure of the 3Gd alloy: (**a**) BF TEM image of secondary phase on triple boundary junctions; (**b**) corresponding SAED pattern of (**a**); (**c**) BF TEM of ellipsoid secondary phase; (**d**) corresponding SAED pattern of (**c**); (**e**) BF TEM image of rod-like secondary phase; (**f**) corresponding SAED pattern of (**e**).

We further studied the microstructures of the 3Gd alloy through TEM determinations for the morphology and crystallography. Figure 3a,c,e display the bright-field (BF) image of network fishbone-shaped secondary phase on triple boundary junctions, network ellipsoidal-shaped secondary phase, and rod-like secondary phase in the interior of the grains, respectively. Their corresponding selected area electron diffraction (SAED) patterns are shown in Figure 3b,d,f, with the electron beams parallel to [0 1 1], [1 2 0], and [1 3 1]. In general, the SAED verifies the type of face-centered cubic (fcc) (Mg, Zn)_3_Gd structure phase for these three microstructures. The lattice parameters are determined to 0.7247, 0.7241, and 0.7301 nm, respectively, smaller than the nominal value of 0.7324 nm for the Mg_3_Gd [21]. Moreover, small variations were observed in the lattice parameters of (Mg, Zn)_3_Gd phase subjected to the Zn and Gd contents [22]. The lattice parameter of (Mg, Zn)_3_Gd phase was significantly reduced with the Zn content in the secondary phase.

**Figure 4:**
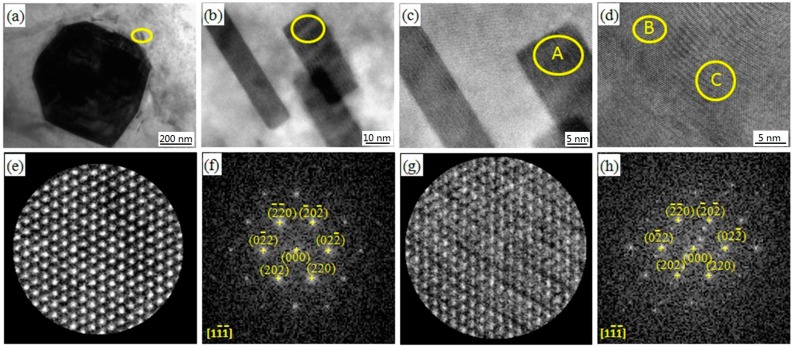
TEM images of the 3Gd alloy: (**a**–**c**) BF TEM image; (**d**) HRTEM image; (**e**) HRTEM image of the B zone; (**f**) FFT image of (**e**); (**g**) HRTEM image of the C zone; (**h**) FFT image of (**g**).

The submicrometer secondary phase was also found in the 3Gd alloy, as shown in Figure 4a. The microsized block (Mg, Zn)_3_Gd phase is surrounded by a small number of nanoscale rod-like secondary phases (marked by yellow ellipse) of 30–50 nm length and 10–15 nm width (Figure 4b). The secondary phase rods oriented in parallel in Figure 4b,c. Figure 4d depicts a high-resolution transmission electron microscope (HRTEM) image taken from the A zone in Figure 4c. An atomic regular arrangement (B zone) and the layered structure (C zone) were observed. The zoomed images of two structures are shown in Figure 4e,g, with their fast Fourier transformed (FFT) results in Figure 4f,h, respectively. The secondary phase marked at B and C zones in Figure 4d was identified as (Mg, Zn)_3_Gd phase with their axes parallel to [1 1 1] direction, as given by FFT results. In comparison with Figure 4f, however, several redundant diffraction spots were found in Figure 4h. This is probably due to lamellar defects in the phase, as shown in Figure 4g.

**Figure 5:**
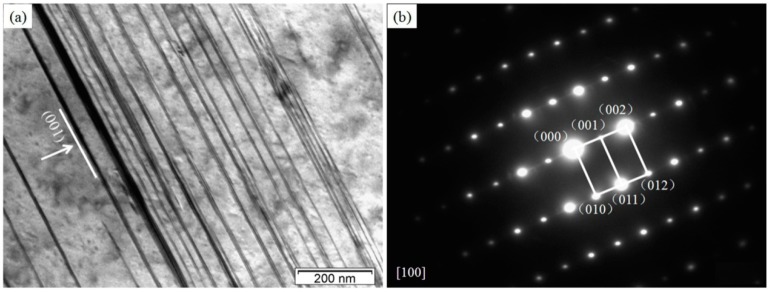
TEM images of the 4Gd alloy: (**a**) BF TEM image of the long period stacking ordered (LPSO) phase; (**b**) SAED pattern corresponding to the LPSO phase.

The LPSO structure turns out in the alloys with higher Gd contents ≥3.0%. The BF TEM images of this structure and the corresponding SAED pattern are given in Figure 5a,b. Parallel to (100) plane, the LPSO structure has a hexagonal close-packed crystal structure as indexing to the diffraction pattern. Lu et al. [23] found a similar LPSO structure in the as-cast Mg_97.1_Gd_1.8_Zn_1_Zr_0.1_ (at %) alloy, and indexed it to the 14H-LPSO structure. Origin of this phase in the (3–5)Gd alloy can be contributed to the slow cooling process during the casting in the preheated steel mold. The layered 14H-LPSO structure has enough time for nucleation and growth, due to the slow cooling rate. This leads to sufficient Zn and Gd atomic diffusion in the matrix. The 14H-LPSO content increases with the Gd percentage, but becomes negligible below the critical percentage of 3%. As reported, the 14H-LPSO phase can improve the thermal stability, elastic modulus, microhardness, and ductility [24]. Besides, the corrosion resistance of Mg alloy can also be enhanced, exhibiting a uniform corrosion morphology [25]. Thus, a moderate amount of 14H-LPSO structure may be beneficial for the materials properties of the alloys.

**Figure 6:**
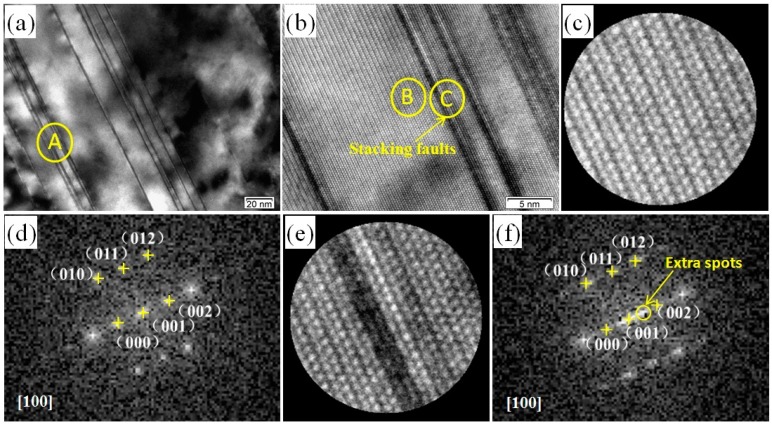
TEM images of 2Gd alloy: (**a**) BF TEM image; (**b**) HRTEM image; (**c**) HRTEM image of the B zone; (**d**) FFT image of (**c**); (**e**) HRTEM image of the C zone; (**f**) FFT image of (**e**).

The microscopic determinations were also carried out for low-Gd content alloys. As an example, the 2Gd alloy was investigated by TEM and HRTEM images. A zoomed image of region A in Figure 6a is given in Figure 6b. The B and C areas in Figure 6b denote the matrix part and the stacking fault structure, respectively. The atomic resolution images of the A and B regions are shown in Figure 6c,e. The regular 2H-Mg structure in Figure 6c clearly prove the well-oriented matrix atoms. However, in the black strip at Figure 6e, an obvious fault phenomenon is detected. To understand this, the FFT to the HRTEM images were studied, in detail, for these two structures (Figure 6d,f). The B and C zones in Figure 6b were all identified as close-packed hexagonal structures, with their zone axis parallel to [100] direction according to its corresponding FFT results. Some tiny irregular SAED spots are seen between the (000) and (002) spots, and attributed to stacking faults. Such structure mainly exists in the alloys of Gd contents below 3.0 wt %, and can be attributed to inadequate diffusion of the Gd and Zn atoms in the Mg matrix. The small quantity of Gd atoms are insufficient to construct the 14H-LPSO structure [26,27].

### 3.2. Mechanical Properties

**Figure 7:**
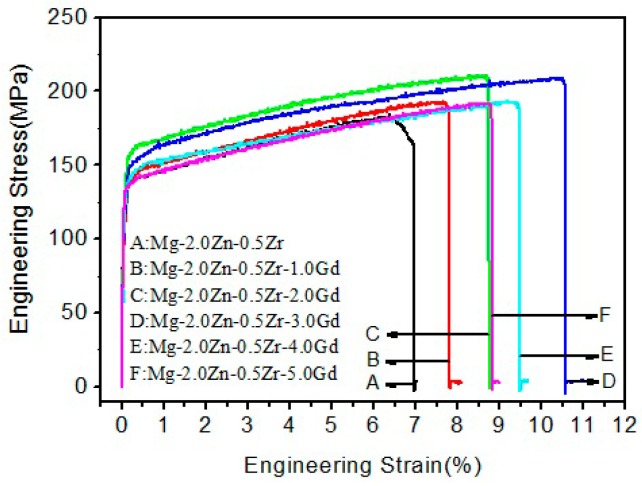
Engineering stress–strain curves at room temperature of the Mg–2.0Zn–0.5Zr–xGd alloys.

Figure 7 plots room temperature stress–strain curves of the as-cast Mg–2.0Zn–0.5Zr–xGd (x = 0–5.0) alloys. The ultimate tensile strength (UTS), yield strength (YS), and elongation to failure (EL) are tabulated in Table 1. In general, the mechanical properties of the alloys have been improved after the Gd addition. The UTS, YS, and EL increase, peak at 3.0 wt %, and then decrease. The better numbers of UTS, YS, and EL elongation at 3Gd are 204 ± 3 MPa, 155 ± 3 MPa, and 10.6 ± 0.6%, respectively.

For Mg–2.0Zn–0.5Zr–xGd alloy, the change of mechanical properties is mainly attributed to solid-solution strengthening, fine-grained strengthening, and distribution of the second phase. After Zn, Zr, and Gd are added to the Mg metal, some of them will dissolve into the α-Mg matrix to form a solid solution. The difference in atomic radius between the alloy elements and magnesium will cause lattice distortion. However, alloying elements interact with the dislocation stress field and lead to solid solution strengthening. As a result, the mechanical properties are enhanced. The differences of valances (electronegativity) also exist among the Zn, Zr, Gd guest atoms and the Mg matrix. This may lead to charge transfers among bonded atoms, and benefits solid-solution strengthening [28,29]. 

The fine grain strengthening is also an important factor for property enhancement. The optimized mechanical properties happens at 3 wt % of the Gd, where the sample has a small grain size (Figure 1). The precipitation strengthening effect of the second phase particles also changes the interfacial energy of matrix. The second phase in small particle size interacts with the matrix via semi-coherent or coherent interfaces [30]. Under this condition, the second-phase particle pinning dislocations inhibit the dislocation motion and result in the strengthening effect. The strength and elongation of the alloy increased gradually under the combined action of refinement strengthening and second phase strengthening [31]. Above 3 wt % Gd load, the (Mg, Zn)_3_Gd phase owns larger grains, more trigeminal boundary, and coarser edges, thus, leading the serious microsegregation. The large FCC-phased (Mg, Zn)_3_Gd structure is incoherent with the α-Mg matrix, weakening the atomic binding force between the (Mg, Zn)_3_Gd and α-Mg [32]. These phases easily induce cracks and their propagations under tension. The strength and plasticity are decreased correspondingly.

### 3.3. Fracture Characteristics

**Figure 8:**
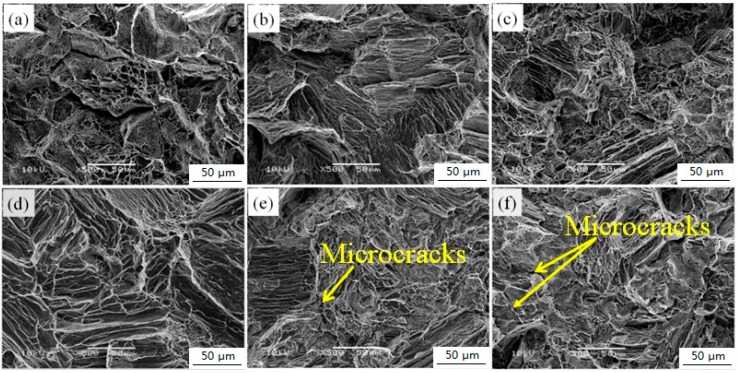
SEM images of fracture surfaces of Mg–2.0Zn–0.5Zr–xGd alloys: (**a**) x = 0; (**b**) x = 1; (**c**) x = 2; (**d**) x = 3; (**e**) x = 4; (**f**) x = 5.

Figure 8 shows the fracture morphology of as-cast Mg–2.0Zn–0.5Zr–xGd (x = 0–5.0) alloys at room temperature. The cleavage surface is perpendicular to the tensile direction for the alloy without Gd content, as shown in Figure 8a. Along the cleaved surface, the typical brittle fracture behaves as the intergranular fracture. At lower Gd content (1.0–3.0 wt %), a large number of tearing edges stretch to different directions, as shown in Figure 8b–d. This is caused by the difference in grain orientation, in the fracture with some small shallow dimples belonging to the hybrid fracture feature of quasi-cleavage fracture and ductile fracture. The elongation of 1.0–3.0 wt % Gd is relatively high, and is consistent with the tensile test results of Table 1. However, at the 4.0–5.0 wt % range, while the cleavage surface area increase gradually with the Gd content, a small number of microcracks appear along the grain boundary (Figure 8e,f). This is attributed to deterioration of the homogeneity and binding forces to the grain boundaries subjected to a large number of coarse second phases [33]. Consequentially, both strength and ductility of the alloy decrease at the range of 4.0–5.0 wt % Gd.

### 3.4. Corrosion Behavior

**Figure 9:**
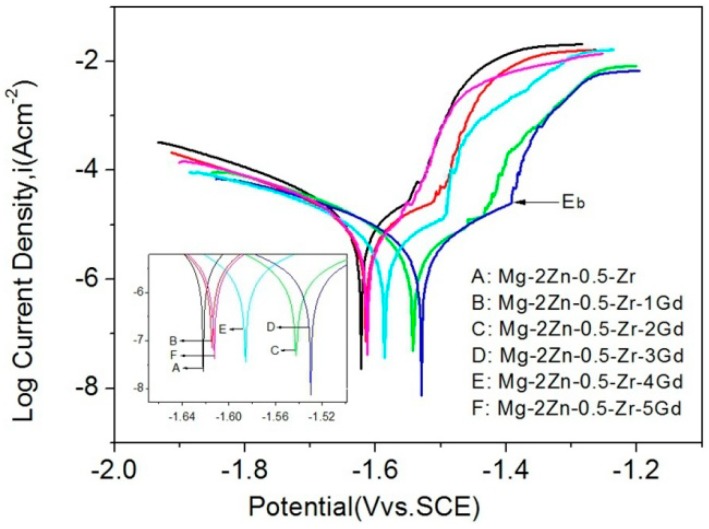
Polarization curves of the Mg–2.0Zn–0.5Zr–xGd alloys.

**Figure 10:**
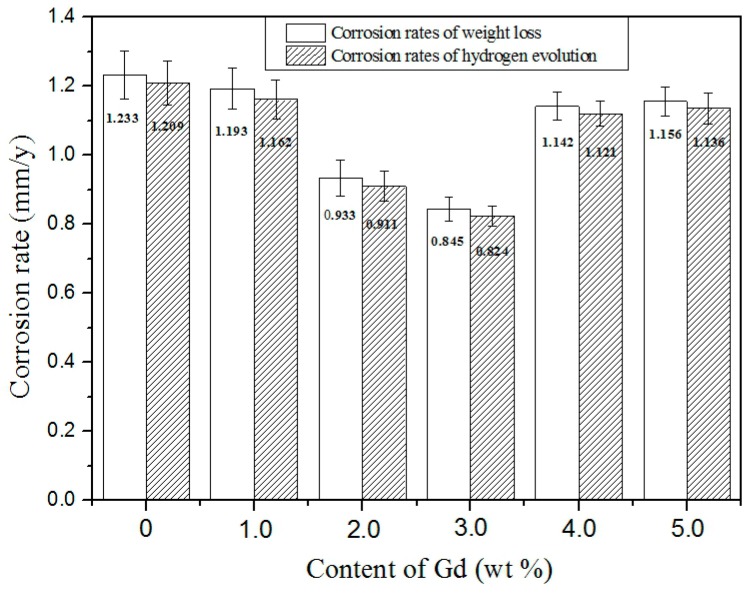
Corrosion rates for Mg–2.0Zn–0.5Zr–xGd alloys measured by weight loss and hydrogen evolution for duration of 120 h.

The corrosion behavior of the alloys was studied and discussed in this subsection. First, the polarization curves obtained for the Mg–2.0Zn–0.5Zr–xGd alloys after 1 h immersion are studied in details. They are plotted in Figure 9. Table 2 tabulates corrosion potentials (*E_corr_*), corrosion current densities (*I_corr_*), and corrosion rates (*P_i_*) deduced from the polarization curves. Figure 10 shows the corrosion rates for the Mg–2.0Zn–0.5Zr–xGd alloys measured by weight loss and hydrogen evolution for a duration of 120 h. At the end of this subsection, the corrosion surfaces were examined to crosscheck the proposed corrosion mechanisms.

As shown in the polarization curves, the *E_corr_* gradually shifted to positive direction, reached its maximum at 3 wt % of Gd, and then decreased. The trends of *I_corr_* and *P_i_* are opposite to the one of *E_corr_*. The *E_corr_*, *I_corr_*, and *P_i_* of the 3 wt % Gd alloy were determined to −1.531 ± 0.003 V vs. SCE, 7.686 ± 0.004 μA cm^-2^, and 0.343 ± 0.002 mm/y. The anodic polarization and cathodic polarization curves represent the dissolution of Mg and the cathodic hydrogen evolution, respectively. A plateau was observed in the polarization curves, indicating a passive film formation on the alloy surface in SBF immersion. Among the as-cast alloys, the 3Gd owns the best corrosion resistivity with the highest positive breaking potential (*E_b_*) and lowest probability of localized corrosion [34]. Indeed, in the Mg–2.0Zn–0.5Zr–xGd alloys, the (Mg, Zn)_3_Gd phase acts as cathode, and α-Mg matrix the anode. Thus, the uniformity of the (Mg, Zn)_3_Gd phase and microstructure refinement of the alloy played an important role in the corrosion resistance of Mg–2.0Zn–0.5Zr–xGd alloys at lower contents ≤3.0 wt %. Grains with smaller sizes serve more boundaries and enhancement of the corrosion resistance thanks to the intensive physical barriers set up by the boundaries [35]. According to the Figure 9 and Table 2, the alloys containing 1.0, 2.0, and 3.0 wt % Gd exhibited lower current densities in comparison with the Gd-free sample. This indicates the beneficial impacts by adding the Gd element (up to 3.0 wt %). On the other hand, the excessive amount of Gd content (>3.0 wt %) results in noticeably detrimental corrosion behavior, where the 4.0 and 5.0 wt % alloys showed considerably higher *P_i_*. Such influences are related to the microstructures of the alloys. In Figure 1, the addition of Gd in higher values led to the formation of interconnected networks of (Mg, Zn)_3_Gd phase in these alloys. The networks of (Mg, Zn)_3_Gd phase becomes thicker as the Gd content increases from 3.0 to 5.0 wt %. In addition, the (Mg, Zn)_3_Gd phase is introduced as local cathodes. In the case of forming many galvanic microcells with α-Mg background, these secondary phases intensely increase the corrosion currents of the alloys with Gd content increases from 3.0 to 5.0 wt %.

Figure 10 shows the corrosion rates for the Mg–2.0Zn–0.5Zr–xGd alloys measured by weight loss and hydrogen evolution for duration of 120 h. The corrosion rate trends given by these two methods were found similar to that measured by Tafel extrapolation as shown in Table 2. Due to hydrogen dissolution in the SBF which overflowed through the container seal, values given by hydrogen evolution were lower than the weight loss counterpart [36]. The corrosion rates gradually decreased following the Gd content from 0 to 3.0 wt %, and then increased from 3.0 to 5.0 wt %. Minimum corrosion rates of 3Gd alloy were determined to 0.845 ± 0.035 mm/y and 0.824 ± 0.031 mm/y, respectively, in weight loss and hydrogen evolution methods.

**Figure 11:**
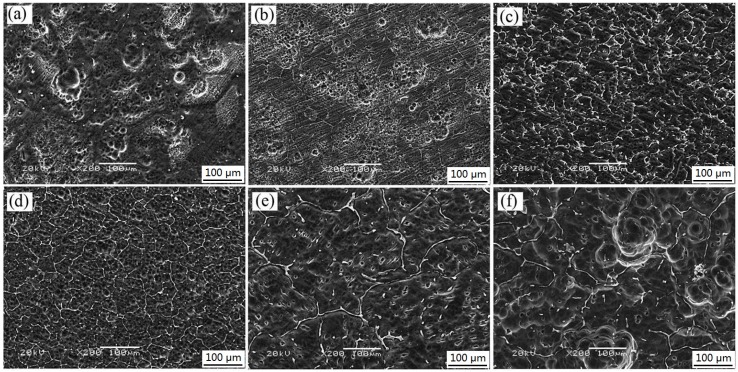
SEM micrographs showing the corroded Mg–2.0Zn–0.5Zr–xGd samples after immersion for 120 h and corrosion product removal: (**a**) x = 0; (**b**) x = 1; (**c**) x = 2; (**d**) x = 3; (**e**) x = 4; (**f**) x = 5.

Figure 11 presents the surface appearance after removal of the corrosion products. At 0 wt % and 1.0 wt % of the Gd contents, samples exhibited a lower density and depth of corrosion pits as shown in Figure 11a,b. This discloses that some pitting corrosion occurred in the alloys. The Gd content has a pronounced influence on the corrosion resistance of the alloys. The occurrence of pitting corrosion is mainly attributed to the discontinuous distribution of the secondary phase along the grain boundaries at the low contents as shown in Figure 1a,b. When the alloys contain 2.0 wt % and 3.0 wt % Gd, samples show smoother corroded surface with less shallow pits (Figure 11c,d). In general, the corrosion type can be justified to the uniform corrosion for these alloys. This type of corrosion mainly arises from the continuous distribution of the second phases along the grain boundaries in the alloys, clearly denoted in Figure 1c,d. The secondary phases in 3Gd alloy formed a uniform and fine network surrounding the α-Mg matrix as shown in Figure 11c,d. Moreover, some alloying elements dissolved into the α-Mg matrix near the network line, yielding the matrix potential increase, and the corrosion resistant enhancement. A certain amount of network secondary phase acts as the barrier and enhances passivation, which inhibited corrosion of the α-Mg matrix. When Gd contents were tuned to 4.0 and 5.0 wt %, the volume fraction of the secondary phase as the cathode increased with the Gd addition in the alloys. These secondary phases deteriorated the corrosion resistance of Mg alloys due to the accelerated micro-galvanic effect [37]. The rapid corrosion of the α-Mg matrix around the some small second phase particles repelled the particles. Consequentially, small holes turned out in Figure 11e,f.

## 4. Conclusions

In conclusion, influence of the Gd content on microstructures, mechanical properties, and corrosion behaviors of as-cast Mg–2.0Zn–0.5Zr–xGd biomedical magnesium alloys were investigated systematically within the work. The following aspects have been extensively studied here.

The as-cast Mg–2.0Zn–0.5Zr–xGd alloys were mainly comprised of α-Mg and (Mg, Zn)_3_Gd phases. With the Gd content (1.0–3.0 wt %) increase, the grain size was obviously refined, and the (Mg, Zn)_3_Gd phase content gradually increased. The alloy corrosion resistance is improved gradually by decreasing the grain size and positively changing the self-corrosion potential. When the Gd content was in the range of 3.0–5.0 wt %, the (Mg, Zn)_3_Gd phase presented a closed network microstructure along the grain boundary. Meanwhile 14H-LPSO phase began to appear in the alloy, and the content of 14H-LPSO phase gradually increased with increasing Gd content. The increasing (Mg, Zn)_3_Gd phase volume at grain boundary increased the corrosion current density between galvanic corrosion, then, the alloy corrosion resistance decreased gradually. When the Gd content was in the range of 0–3.0 wt %, combining the fine crystal strengthening and the second phase strengthening made the alloy mechanical properties gradually increase with increasing Gd content. When the Gd content was in the range of 3.0–5.0 wt %, with the Gd content increases, eutectic phase distributed along the grain boundary which weakened the intercrystalline bonding, and the alloy mechanical properties gradually decreased. The alloy at 3.0 wt % Gd has good corrosion resistance and mechanical properties, whereby the corrosion rates are 0.845 ± 0.035 mm/y and 0.824 ± 0.031 mm/y, as given by weight loss and hydrogen evolution immersing in SBF at 37 °C for 120 h, respectively. The UTS, YS, and EL are 204 ± 3 MPa, 155 ± 3 MPa, and 10.6 ± 0.6%, respectively.

## Figures and Tables

**Table 1:** Mechanical properties of Mg–2.0Zn–0.5Zr–xGd alloys.

Material	0Gd	1Gd	2Gd	3Gd	4Gd	5Gd
UTS (MPa)	183 ± 3	193 ± 4	210 ± 3	204 ± 3	196 ± 4	192 ± 3
YS (MPa)	137 ± 3	143 ± 4	158 ± 3	155 ± 3	146 ± 4	141 ± 3
EL (%)	7 ± 0.6	7.8 ± 0.5	8.7 ± 0.6	10.6 ± 0.6	9.5 ± 0.5	8.9 ± 0.5

**Table 2:** *E_corr_*, *I_coor_*, and *P_i_* of Mg–2.0Zn–0.5Zr–xGd alloys derived from the polarization curves.

Material	0Gd	1Gd	2Gd	3Gd	4Gd	5Gd
*E_corr_* (Vvs.SCE)	−1.621 ± 0.004	−1.615 ± 0.003	−1.542 ± 0.004	−1.531 ± 0.003	−1.586 ± 0.003	−1.613 ± 0.004
*I_corr_* (μA cm^−2^)	9.278 ± 0.005	8.947 ± 0.004	8.136 ± 0.005	7.686 ± 0.004	8.746 ± 0.004	8.863 ± 0.005
*P_i_* (mm/y)	0.416 ± 0.003	0.401 ± 0.002	0.367 ± 0.003	0.343 ± 0.002	0.389 ± 0.002	0.398 ± 0.003
